# Brain fingerprinting classification concealed information test detects US Navy military medical information with P300

**DOI:** 10.3389/fnins.2014.00410

**Published:** 2014-12-23

**Authors:** Lawrence A. Farwell, Drew C. Richardson, Graham M. Richardson, John J. Furedy

**Affiliations:** ^1^Brain Fingerprinting Laboratories, Inc./Brain Fingerprinting, LLCSeattle, WA, USA; ^2^Federal Bureau of Investigation, FBI LaboratoryQuantico, VA, USA (at the time of the research); ^3^Department of Cell and Developmental Biology, Vanderbilt UniversityNashville, TN, USA; ^4^Department of Psychology, University of TorontoToronto, ON, Canada

**Keywords:** P300, concealed information test, brain fingerprinting, P300-MERMER, ERP, LNP, event-related potential, detection of concealed information

## Abstract

A classification concealed information test (CIT) used the “brain fingerprinting” method of applying P300 event-related potential (ERP) in detecting information that is (1) acquired in real life and (2) unique to US Navy experts in military medicine. Military medicine experts and non-experts were asked to push buttons in response to three types of text stimuli. Targets contain known information relevant to military medicine, are identified to subjects as relevant, and require pushing one button. Subjects are told to push another button to all other stimuli. Probes contain concealed information relevant to military medicine, and are not identified to subjects. Irrelevants contain equally plausible, but incorrect/irrelevant information. Error rate was 0%. Median and mean statistical confidences for individual determinations were 99.9% with no indeterminates (results lacking sufficiently high statistical confidence to be classified). We compared error rate and statistical confidence for determinations of both information present and information absent produced by classification CIT (Is a probe ERP more similar to a target or to an irrelevant ERP?) vs. comparison CIT (Does a probe produce a larger ERP than an irrelevant?) using P300 plus the late negative component (LNP; together, P300-MERMER). Comparison CIT produced a significantly higher error rate (20%) and lower statistical confidences: mean 67%; information-absent mean was 28.9%, less than chance (50%). We compared analysis using P300 alone with the P300 + LNP. P300 alone produced the same 0% error rate but significantly lower statistical confidences. These findings add to the evidence that the brain fingerprinting methods as described here provide sufficient conditions to produce less than 1% error rate and greater than 95% median statistical confidence in a CIT on information obtained in the course of real life that is characteristic of individuals with specific training, expertise, or organizational affiliation.

## Introduction

### The classification CIT

The concealed information test (CIT) or guilty knowledge test (GKT) has been used to detect concealed information since Lykken (1959). Until the 1980s, the dependent measures were autonomic nervous system (ANS) responses. The ANS-based CIT is a *comparison* CIT (Lykken, 1959). The comparison CIT compares the responses to crime- or situation-relevant and irrelevant items. If the responses to the relevant items are larger, then the determination is made that the subject knows the relevant information. (“Larger” is variously defined.) Otherwise, the determination is made that the subject does not know the information.

Farwell and Donchin (1991) introduced three innovations in the CIT (Farwell, 2013). They (1) applied a classification CIT, rather than the conventional comparison CIT; (2) used event-related brain potentials (ERPs) as the dependent measure; and (3) computed a statistical confidence for each individual determination using the technique of bootstrapping. (Farwell and Donchin, 1991 was preceded by abstracts on the same studies, Farwell and Donchin, 1986, 1988a). Several researchers subsequently applied ERPs and bootstrapping in a comparison CIT (e.g., Johnson and Rosenfeld, 1992; Rosenfeld et al., 2004, 2008; Meixner and Rosenfeld, 2014). This is a fundamentally different paradigm (see Discussion and Appendix 2).

In the classification CIT, three types of stimuli are presented. (1) “Probes” are relevant to the investigated situation. Probes contain information that the subject has no way of knowing other than participation in the investigated situation (and, in field cases, that the subject denies knowing or recognizing as being crime-relevant). (2) “Targets” also may be relevant to the investigated situation. (In all our recent applications including the study reported here, they are.) Experimental protocols ensure that the subject knows the targets, for reasons other than participation in the investigated situation. The response to targets provides a template for the subject's response to known, situation-relevant information. (3) “Irrelevants” contain irrelevant information. The response to irrelevants provides a template for the subject's response to irrelevant information. If the ERP response to the probes is mathematically classified as being more similar to the ERP response to the known, relevant target information than to the irrelevants, the subject is determined to be “information present” with respect to the information contained in the probes. If the ERP response to the probes is more similar to the ERP response to the irrelevant information than to the targets, the subject is determined to be “information absent.” If the probe ERP response cannot be classified with a high statistical confidence as being more similar to either the target or the irrelevant response, no determination is made; the outcome is “indeterminate.”

The classification CIT with ERPs can be applied in two different types of tests. Specific issue tests detect knowledge of a specific event such as a crime. Specific screening or focused screening tests detect knowledge relevant to specific training or expertise, or inside knowledge of a particular organization or group. This study is a specific screening test conducted in collaboration with the US Central Intelligence Agency (CIA) and the US Navy. The information detected is military medical knowledge in US Navy military medical experts.

We compared the classification CIT with the comparison CIT by analyzing our data with both methods. Farwell and Donchin (1991) used the classification CIT with the P300, an electrically positive event-related potential (ERP) maximal at the parietal midline area of the head that is elicited when a subject recognizes and takes note of a stimulus that is significant in the current context. In this research we compared the results of using the P300 alone vs. the P300 plus the late negative component (LNP; together, P300-MERMER, memory and encoding related multifaceted electroencephalographic response)^1^. We compared error rate and statistical confidences produced by the classification CIT with the results of analysis applying the comparison CIT on the same data. We investigated whether the classification CIT provides significantly lower error rate and higher statistical confidences than the comparison CIT.

### Previous research on sufficient and necessary conditions for a viable CIT for real-world field use

In our view, in order to be considered reliable, an ERP-based CIT must reliably produce less than 1% error rate and median statistical confidences of greater than 95% for individual determinations, including median statistical confidences greater than 90% for both information-present and information-absent determinations, across different field and laboratory conditions. These same criteria, in our view, are the minimum criteria required to effectively and ethically apply a technique in criminal investigations or any application with non-trivial consequences.

Farwell and Donchin's (1991) method provided the sufficient conditions to meet these requirements. They only established sufficient conditions, and did not investigate which conditions were necessary. Since then, research has progressed substantially in two parallel, largely non-overlapping series of studies. One series of studies has investigated the sufficient conditions to meet these criteria under varying field and laboratory conditions. Another series of studies has investigated the necessary conditions.

Eight previous peer-reviewed studies conducted by six researchers in four laboratories have applied a specific set of methods in the ERP-based CIT [Farwell and Donchin, 1991 (two studies); Allen and Iacono, 1997; Farwell and Smith, 2001; Farwell et al., 2013 (four studies); see also Iacono, 2008]. These specific methods are the only methods that have reliably produced less than 1% error rate and median 95% statistical confidences for individual determinations, including over 90% for both information-present and information-absent determinations, in the laboratory and the field. These same error rates and statistical confidences have been achieved with countermeasures, without countermeasures, and in field conditions where it is unknown whether countermeasures are being used or not (Farwell et al., 2013). (Countermeasures are physical or mental procedures that a subject may practice in an attempt to influence the outcome of a test. They were not studied in this research).

The methods applied in these studies are the same as in the original Farwell and Donchin studies, with several improvements based on the more demanding requirements of field applications, as described below. Farwell (2012) documented these methods (or rules or recommendations) as 20 brain fingerprinting scientific standards (Appendix 1). Farwell (1994, 2012) defined “brain fingerprinting” as the classification-CIT technique incorporating the 20 standards. These methods are applied here^2^. We have focused our previous research primarily on establishing the sufficient conditions because this provides a technique we can use, and have successfully used, in the field. Previous studies by others investigated the necessary conditions to obtain low error rates and high statistical confidences (Farwell, 2012, 2014; Appendix 2).

### P300 and LNP

Farwell and Donchin (1991) used the classification CIT with the P300. In this research we compared the results of using the P300 alone vs. the P300 plus the LNP. Our rationale for this is as follows.

Early P300 research (e.g., Sutton et al., 1965) used very simple stimuli, such as auditory clicks and tones. As the sophistication of experimental designs progressed, more complex stimuli were used, including simple words and phrases presented visually. The latency of P300 was found to increase with stimulus complexity and the concomitant stimulus evaluation time (Magliero et al., 1984). With simple words and phrases, an inter-stimulus interval (ISI) of 1000–1500 ms or less was adequate for the subject to process the stimuli and to capture the entire ERP response (Farwell and Donchin, 1988b). Farwell and Donchin (1991), for example, used phrases consisting of two, one-syllable words, and an ISI of 1500 ms.

In conducting research at the FBI in 1993, however, Farwell et al. (2013; Farwell, 1994, 1995a,b) had the task of developing text stimuli that accurately represented knowledge unique to FBI agents. This required some stimuli to be several long words. To give subjects time to fully process the stimuli, we extended the ISI to 3000 ms. Under these conditions, we found that the positive P300 peak was followed by a negative peak with a peak latency of up to 1200 ms, which we termed the late negative potential (LNP).

The stimuli we used in this study and in previous research (Farwell and Smith, 2001; Farwell et al., 2013) were more personally significant than the stimuli presented in most previous P300 research. LNP may be driven at least in part by this personal significance. Compared to many previous P300 studies, our stimuli may also be more salient, be more related to previous memories, require more complex processing, and involve a task more important to the subject. They are also presented with a longer ISI than that applied in most previous P300 studies. Further research is necessary to identify the antecedent conditions and delineate the functional significance of the LNP.

We called the overall pattern of the P300 followed by the LNP in the time domain, along with concomitant changes in the frequency domain and other putative changes measurable by other mathematical methods, a P300-MERMER. Although the P300-MERMER—and for that matter, the P300—may be comprised of additional features that are not visible in the time domain (Farwell, 2012; Farwell et al., 2013), the time-domain pattern is sufficient to define and to detect the response. This pattern consists of a positive peak followed by a negative peak (or a negative-positive-negative pattern if the N200, a well-known negative component that generally precedes the P300, is included).

We compared results obtained using the P300 alone with results obtained by including the P300 plus the LNP. Our computations consider only the conventional, time-domain characteristics of the signals. The difference between our two epoch-related analyses is the length of the epoch analyzed, and therefore the inclusion or exclusion of the LNP and its amplitude, morphology, and latency.

In the early 1990s, when Farwell et al. (2013) first encountered the LNP that follows the P300, we initially hypothesized that LNP was an artifact, perhaps generated by the analog filters and the return of the P300 to baseline. The data contradicted this hypothesis, however. If the LNP were an artifact produced by the filter's effect on the P300, then similar P300s with identical filters would produce similar LNPs. We found that the latency, amplitude, and morphology of the LNP varied independently of the P300. Also, the scalp distribution of the LNP was more frontal than that of the P300. Moreover, the negative peak persisted when we varied our filter settings (Farwell, 1994, 2012). Even recording without analog filters did not eliminate the LNP, or substantially change its characteristics. This definitively disproved the filter-generated-artifact hypothesis (Farwell et al., 2013).

The data we recorded with filters also contradict the hypothesis that the LNP is an artifact. We used the same recording equipment for all subjects and all scalp sites. If the LNP were an artifact produced by the equipment, the same equipment would produce identical effects in different scalp sites and different subjects. The features of the LNP would be a function of the features of the P300. This was not observed. For different scalp sites in one subject, and for different subjects, the relative amplitude, latency, and morphology of the LNP and the P300 were very different. Sometimes there was a difference of hundreds of milliseconds in the latency, and amplitude differences of a factor of two or more, in LNPs that followed virtually identical P300s recorded from different subjects (Farwell et al., 2013). In some cases the LNP was considerably larger than the P300 at one scalp site (Fz) and considerably smaller than the P300 at another (Pz) for the same subject. In short, the data contradict the hypothesis that the LNP (or the latter part of the P300-MERMER) is an artifact produced by some combination of the P300, the return to baseline after the P300, and the filters and other equipment.

In the current paradigm, a negative peak (the N200) precedes the P300 positive peak, and another negative peak (the LNP) follows the P300. Our first observation of this tri-phasic negative-positive-negative morphology in the ERP response was in the early 1990s (Farwell, 1994, 2012; Farwell and Smith, 2001; Farwell et al., 2013). Others applying intracranial recordings have observed this same negative-positive-negative pattern in a number of brain structures (Halgren et al., 1998; Linden, 2005). These include dorsolateral and orbital frontal cortices, anterior cingulate (Baudena et al., 1995), amygdala and hippocampus (Halgren et al., 1986; Stapleton and Halgren, 1987), superior temporal sulcus (Halgren et al., 1995), and inferior parietal lobe/supramarginal gyrus (Smith et al., 1990).

Others investigating the ERP-based CIT, including Meijer et al. (2007), have also reported the LNP. Brouwer et al. (2010) observed the LNP and investigated its utility in brain-computer interfaces. Several other studies (Matsuda et al., 2009; Gamer and Berti, 2010, 2012) reported a difference in the N200 in responses to relevant stimuli in ERP-based CITs. Virtually all researchers conducting research on ERP-based CITs now include in their data-analysis algorithms both the P300 and the LNP (for reviews, see Farwell, 2012, 2014), although some refer to the entire response including both positive and negative peaks as “P300” (e.g., Rosenfeld et al., 2008) and some refer to the positive peak as “P300” and the entire response as “P300-MERMER” or “P300 + LNP” (e.g., Sutton et al., 1965; Farwell, 2012, 2014; Farwell et al., 2013).

Changes in the frequency domain and other changes in the dimensionality and other characteristics of the signal may be included in the term “P300-MERMER.” The positive and negative time-domain changes constituting the P300 and the LNP are sufficient to detect and characterize the response, and are all that are measured in this research, although they undoubtedly do not constitute a complete and comprehensive description of all the patterns of electrophysiological activity that manifest the underlying information-processing brain activity (Farwell, 1994, 2012; Farwell and Smith, 2001).

We compared the error rate and statistical confidences produced by data analysis including the P300 plus the LNP with the results of analysis using the P300 alone. We investigated whether the classification-CIT analysis with the P300 plus the LNP provides significantly lower error rate and/or higher statistical confidences than the analysis with the P300 alone.

### Summary of research questions

Our primary research questions are as follows:

1. Does the classification CIT provide significantly (a) lower error rate and/or (b) higher statistical confidences than the comparison CIT.
2. Does the classification-CIT analysis with the P300 + LNP provide significantly (a) lower error rate and/or (b) higher statistical confidences than the analysis with the P300 alone.

## Materials and methods

### Subjects

We tested 16 experts (information present) and 14 non-experts (information absent) in military medicine. Experts were students and faculty at Uniformed Services University of the Health Sciences (USUHS) possessing professional knowledge of military medicine. Non-experts lacked this specific expertise and training. Mean age of 30 subjects was 26; standard deviation was 2.9. Mean ages of information-present and information-absent subjects were 27 and 25, respectively; standard deviations were 3.2 and 2.6, respectively. 15 subjects (8 information present) were female.

Experimental procedures were approved by the Brain Fingerprinting Laboratories, Inc., ethics committee and performed in accordance with the ethical standards of the 1964 Declaration of Helsinki, including written informed consent prior to participation.

### Stimuli

Three types of stimuli consisting of words or phrases were presented on a computer screen: probes, targets, and irrelevants. Probes contain specific information relevant to the investigated situation. The test is designed to detect the subject's knowledge or lack of knowledge of the information contained in the probes as relevant in the context of the investigated situation.

In this specific screening study, the relevant information detected was known only to experts in military medicine. Information was obtained from interviews with USUHS military medical experts. Individuals interviewed were not tested. Probe stimuli contained the relevant information to be detected. We presented two additional types of stimuli. Responses to target stimuli provide a template for the subject's brain response to known information relevant to the investigated situation. Responses to irrelevant stimuli provide a template for the subject's brain response to irrelevant information. Target stimuli present information relevant to the investigated situation that is known to be known to the subject. There are significant, proven advantages to using targets that are relevant to the investigated situation rather than inherently irrelevant targets that are made relevant only by task instructions (Farwell, 2012; Farwell et al., 2013), although we and others have successfully used the latter (Farwell and Donchin, 1991). Target stimuli, unlike probes, were identified as such to the subject in experimental instructions. Subject instructions also conveyed the significance of each target in the context of the investigated situation, and required a different behavioral response to targets than to probes and irrelevants, as described in the next section.

For each probe (and each target) comparable irrelevants were structured that contained similar, plausible, but incorrect information about the investigated situation. For a subject lacking the relevant knowledge contained in the probes, the irrelevants and probes were equally plausible as correct, relevant details. Each probe and its comparable irrelevants were indistinguishable for a subject lacking the information that the test was structured to reveal. Each probe contained correct, relevant information fitting the description of that probe. The two irrelevants comparable to each probe contained incorrect information that would be plausible as fitting that same description for an individual lacking the information contained in the probes. For example, a probe stimulus could be the technical name of a military medical procedure in which experts are trained. Corresponding irrelevants could be technical terms that do not name any real procedure. For security reasons, the exact stimuli cannot be given. Subjects were provided with a description of each probe that specified the significance of the probe in the context of the investigated situation, but were not informed which was the correct, situation-relevant probe and which were the corresponding irrelevants.

Similarly, each target stimulus contained correct, situation-relevant information, and the two irrelevant stimuli comparable to each target contained comparable, incorrect but plausible information. Unlike probes, targets were identified as such in instructions to the subjects.

Stimuli were constructed in groups of six: one probe, one target, and four irrelevants. For each probe there were two comparable irrelevants. For each target there were two comparable irrelevants. We used a ratio of 1/6 targets, 1/6 probes, and 2/3 irrelevants so targets and probes were relatively rare, which is known to enhance P300 amplitude (Farwell and Donchin, 1991).

Our prediction was that targets would elicit a large P300 + LNP (or P300-MERMER) in all subjects, irrelevants would not elicit a large P300 + LNP, and probes would elicit a large P300 + LNP only in information-present subjects. Thus, for information-present subjects, ERP responses to probes would be similar to ERPs for targets. For information-absent subjects, ERP responses to probes would be similar to ERPs for irrelevants.

There were 32 unique probes, 32 unique targets, and 128 unique irrelevants, a total of 192 unique stimuli. These comprised 32 groups of stimuli, each consisting of one probe, one target, and four irrelevants. 20 probes were words or phrases embodying the relevant knowledge; 12 were acronyms. The same stimuli were presented to all subjects. Each unique stimulus was presented more than once, so the total number of stimulus presentations was greater than the total number of unique stimuli.

### Procedure

Before the test, we made certain that the subject understood the significance of the probes. We described the significance of each probe to the subject. We then showed the subject the probe and the corresponding irrelevants, in the context of the description of the significance of the probe, without revealing which was the probe. Thus, subjects were informed of the significance of each probe stimulus, but were not told which stimulus was the probe and which were corresponding irrelevants. For example, subjects were told, “One of these three items is the term for a medical technique applied to burn victims in battlefield situations” followed by a list of one probe and two irrelevants (in alphabetical order). Although the descriptions of the probes were made known to subjects, the probe stimuli themselves were never identified as probes.

Targets were explicitly identified to the subjects. Experimental instructions ensured that the subject knew the targets and their significance in the context of the investigated situation. We described the significance of each target to the subject. We showed the subject each target and the corresponding irrelevants, in the context of the description of the significance of the target. We also showed subjects a list of the targets, and noted that subjects would be required to recognize the targets during the test. We instructed subjects to press a button with one thumb in response to targets, and another button with the other thumb in response to “all other stimuli.” The subject's task was to read and comprehend each stimulus, and then to indicate by a button press whether the stimulus was a target stimulus or not.

For a subject possessing the knowledge embodied in the probes, “all other stimuli” consisted of two types of stimuli: probes containing the known situation-relevant information, and irrelevant stimuli. For a subject lacking the tested knowledge, “all other stimuli” appeared equally irrelevant. Probes were indistinguishable from irrelevants. For “all other stimuli” (that is, everything except targets), the subject was instructed to push the opposite button from the one pushed in response to targets. This instruction applied whether the subject perceived these as a single category (all equally irrelevant, if the subject was information absent) or as two categories (irrelevant, and relevant to the concealed information being tested, if the subject was information present).

The differential button-press task in response to every stimulus presentation ensured that the subject was required to read and comprehend every stimulus, including the probe stimuli, and to prove behaviorally that he had done so on every trial. This allowed us to avoid depending on detecting brain responses to assigned tasks that the subject could covertly avoid doing, while performing the necessary overt responses (see Appendix 2).

Testing was divided into separate blocks. In each block the computer display presented 72 stimulus presentations or trials. In blocks 1–3, four stimulus groups were presented in each block, that is, in each block there were four unique probes, four unique targets, and 16 unique irrelevants. Each stimulus was presented three times in a block to make the total of 72 stimulus presentations per block. Stimuli were presented in random order. In blocks 4–7, five stimulus groups were presented in each block in random order until 72 trials had been presented. (Since the total of 72 trials is not divisible by 5, some randomly selected stimuli were presented 3 times and some 4.) In blocks 1–3, stimuli were acronyms. In blocks 4–7, stimuli were words and phrases.

Immediately before each block, we repeated the description of the significance of each of the probes and targets that were to appear in each block (but not the actual stimuli). For example, “In this test you will see the term for a medical technique applied to burn victims in battlefield situations, a medical instrument applied in field wound treatments, a type of injury sustained from exposure to chemical weapons, and the name of the individual who developed the preferred treatment for exposure to sarin gas.”

Stimuli were presented for 300 ms at an ISI of 3000 ms. A fixation point (“X”) was presented for 1000 ms prior to each stimulus. For each trial, the sequence was a fixation point for 1000 ms, the stimulus (target, probe, or irrelevant) for 300 ms, a blank screen for 1700 ms, and then the next fixation point.

Trials contaminated by artifacts generated by eye movements or muscle-generated noise were rejected on-line, and additional trials were presented until 72 artifact-free trials were obtained. Trials with a range of greater than 97.7 microvolts in the EOG channel were rejected. Data for “rejected” trials were collected and recorded, but rejected trials did not contribute to the count of trials presented, so each rejection resulted in an additional stimulus presentation. In 7 blocks, a total of 84 probe, 84 target, and 336 irrelevant artifact-free trials were collected, for a grand total of 504 trials. (Previous research, e.g., Fabiani et al., 1987 has shown that repeating the stimuli does not substantially affect the relevant brain response.)

Brain responses were recorded from the midline frontal, central, and parietal scalp locations (Fz, Cz, and Pz, International 10–20 system) referenced to linked mastoids, and from a location on the forehead to track eye movements. Med Associates silver-silver chloride disposable electrodes were held in place by a custom headband.

Data were digitized at 333 Hz, and resampled at 100 Hz off-line for analysis. Electroencephalograph (EEG) data were amplified at a gain of 50,000 using custom amplifiers. Electro-oculograph (EOG/eye movement) data were amplified at a gain of 10,000. Impedance did not exceed 10 kilohm. Analog filters passed signals between 0.1 and 30 Hz. Data were stored on disk for off-line analysis.

### Data analysis

We analyzed ERP data from the Pz scalp site. Data were digitally filtered using a 49-point, equal-ripple, zero-phase-shift, optimal, finite impulse response, low-pass filter with a passband cutoff frequency of 6 Hz and a stopband cutoff frequency of 8 Hz (Farwell et al., 1993). Trials with a range of greater than 97.7 microvolts in the EOG channel were excluded from analysis. We decided on this threshold based on our previous experience (Farwell and Donchin, 1991; Farwell et al., 2013). In exploratory data analysis, we have varied this threshold considerably, and the results are robust even if we change this parameter within quite a wide range.

For each subject's data we conducted two separate classification-CIT analyses applying bootstrapping as described below. One analysis used the positive P300 peak followed by the LNP, a later negative peak (together also known as the P300-MERMER). A second analysis included only the positive P300. The P300 + LNP epoch was defined as 300–1800 ms after stimulus onset. The P300 epoch was 300–900 ms after stimulus onset. The two analyses were identical except for the epoch analyzed. A third analysis applied bootstrapping with the comparison CIT on the full P300 + LNP epoch, as in previous ERP studies with the comparison CIT.

The data analysis produced three sets of results for each subject: (1) a determination of information present or information absent along with a statistical confidence for the determination using the classification CIT and the full P300 + LNP epoch; (2) a comparable determination and statistical confidence using the P300 alone with the classification CIT; and (3) a comparable determination and statistical confidence using the comparison CIT on the full epoch. This allowed us to compare the error rate/accuracy and statistical confidence provided by (a) the P300 + LNP vs. the P300 alone in a classification CIT, and (b) the classification CIT vs. the comparison CIT.

### Bootstrapping

#### Classification-CIT bootstrapping method

The primary data-analysis task was to determine whether the ERP responses to the probe stimuli contained a large P300 and LNP similar to that elicited by the targets, or whether the probe responses lacked a large P300 and LNP, like the irrelevants.

We used bootstrapping (Wasserman and Bockenholt, 1989; Farwell and Donchin, 1991; Farwell et al., 2013) to determine whether the probe responses were more similar to the target responses or to the irrelevant responses, and to compute a statistical confidence for this determination for each individual subject. The metric for similarity was double-centered correlation.

The bootstrapping procedure accomplished two goals: (1) to take into account the variability across single trials, while also maintaining the smooth and relatively noise-free shape provided by signal averaging; (2) to isolate the critical variable—knowledge of the information embodied in the probes—by classifying the responses to the probe stimuli as being either more similar to the target responses or to the irrelevant responses. We conducted two classification-CIT analyses, one using only the P300 and one using the P300 plus the LNP (together also known as the P300-MERMER).

Briefly, the bootstrapping procedure for the classification CIT is as follows. We repeat the following procedure 1000 times. Randomly sample P probes, T targets, and I irrelevants, with P, T, and I equal to the total number of probe, target, and irrelevant trials in the data set, respectively. In each iteration, compare the probe-target correlation with the probe-irrelevant correlation. Count the number of times that the probe-target correlation is greater than the probe-irrelevant correlation, and convert this to a percentage. This is the probability that the probe response is more similar to the target response than to the irrelevant response, or the probability that information present is the correct determination. 100% minus this is the probability that the probe response is more similar to the irrelevant response, or the probability that information absent is the correct determination.

We set an *a priori* bootstrapping probability criterion of 90% for an information-present determination and 70% (in the opposite direction) for an information-absent determination. If the probability was greater than 90% that the probe response was more similar to the target response than to the irrelevant response, we classified the subject as information present. The bootstrap probability is the statistical confidence for this determination.

The probability that information absent is the correct determination is 100% minus the probability that information present is the correct determination. For example, if there is a 90% probability that the probe response is more similar to the target than to the irrelevant response (information present is correct), then there is a 10% probability that the probe response is more similar to the irrelevant response (information absent is correct). If the probability was greater than 70% that the probe response was more similar to the irrelevant response than to the target response (equivalent to a 30% probability that the probe response was more similar to the target response), we classified the subject as information absent. The bootstrap probability is the statistical confidence for this determination.

If the results did not meet either criterion, we did not classify the subject in either category. The outcome would then be indeterminate (although there were no indeterminates).

For each subject, each data analysis method produced a determination and a statistical confidence, e.g., information present, 99.9% statistical confidence. The statistical confidence is the probability that the determination is correct, based on the within-subjects statistical computation taking into account the size of the effect and the variability in the data.

**Figure 1:**
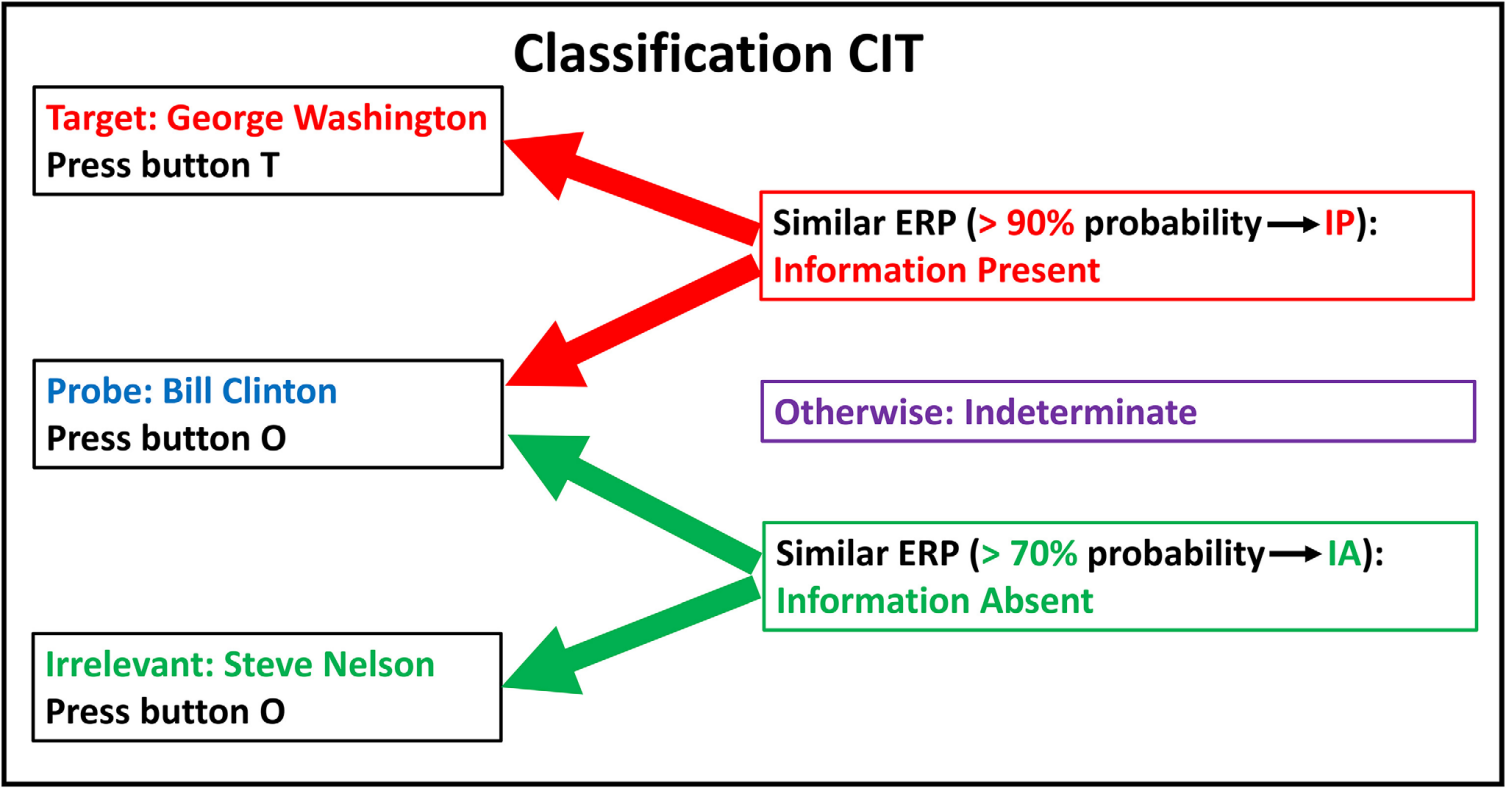
**Classification CIT: stimuli, responses, and determinations**. Example of a classification CIT to determine whether an individual has information about US political history. Subject instructions: “You will view names of former US presidents. Some of these are on this list: George Washington, John Adams, Ronald Reagan. Press button T [target] for any name on this list. Otherwise press button O [other]. George Washington was a US president on the list, so then you should press T.” Since Bill Clinton was not on the list and was not identified to the subject as a US president, the subject will press button O for Bill Clinton, whether he recognizes him as a US president (information present) or not (information absent). IP, Information Present; IA, Information Absent.

Figure 1 illustrates example stimuli, ERP responses, bootstrapping probabilities, and determinations for a hypothetical classification CIT to determine if an individual has information regarding US political history.

Error rate is the percentage of incorrect information-present (false positive) and information-absent (false negative) determinations. Accuracy is 100% minus the error rate. In reporting error rates and/or accuracy, indeterminates must be reported as such. In reporting “accuracy,” some authors have confounded indeterminates with false positives and/or false negatives, reporting “accuracy” as the percentage of tests that result in a correct determination, and hiding the number of indeterminates. This irretrievably hides the true error rate if there are indeterminates, and makes it impossible to make a meaningful comparison with studies that report the true error rate. In any meaningful reporting, indeterminates if any must be identified as such, and not confounded with false positive or false negative errors. (Some legitimate techniques such as Bayesian analysis do not allow indeterminates, in which case this must also be reported).

We restricted our conclusions to a determination as to whether or not a subject knew the specific situation-relevant knowledge embodied in the probes at the time of the test. Our procedures recognize the fact that the ERP-based classification CIT detects only presence or absence of information—not guilt, innocence, honesty, lying, deception, or any past action or non-action.

#### Comparison-CIT bootstrapping method

The comparison CIT uses bootstrapping in an entirely different way. The comparison CIT ignores the target responses and applies bootstrapping to compute the probability that the amplitude of the probe ERP is larger than the amplitude of the irrelevant ERP. The amplitude of the ERP response is defined as the difference between the highest voltage in the P300 window (300–900 ms) and the lowest voltage in the LNP window (900–1800 ms). (This is essentially the sum of the peak amplitudes of the P300 and the LNP.) This is in accord with the metric used in previous applications of the comparison CIT (e.g., Rosenfeld et al., 2008).

Trials are randomly sampled with replacement and averaged as described above for the classification CIT, except that only probe and irrelevant trials are sampled and averaged. In each of 1000 iterations, the amplitude of the ERP in the sampled probe average is compared with the amplitude of the ERP in the sampled irrelevant average. The percentage of times that the sampled probe ERP is larger than the sampled irrelevant ERP provides an estimate of the probability that the probe ERP is larger than the irrelevant ERP. If the probability that the probe ERP is larger than the irrelevant ERP is greater than 90%, then the subject is determined to be information present. If the probability that the probe ERP is larger than the irrelevant ERP is less than 90%, then the subject is determined to be information absent. (The comparison CIT does not have an indeterminate category.) A probability of 90% that information present is correct (that is, probe ERP is larger than irrelevant ERP) is equivalent to a probability of 10% (that is, 100%–90%) that information absent is correct. Therefore, any subject with a probability of over 10% that information absent is correct is determined to be information absent. This results in subjects being determined to be information *absent* when the computed bootstrap probability is as high as 89.9% that information *present* would be the correct determination, that is, as low as a 10.1% statistically computed probability that the selected information-absent determination is correct. Information-absent statistical confidences range from 10.1% to 99.9% and average 50% (chance) (see Appendix 2).

**Figure 2:**
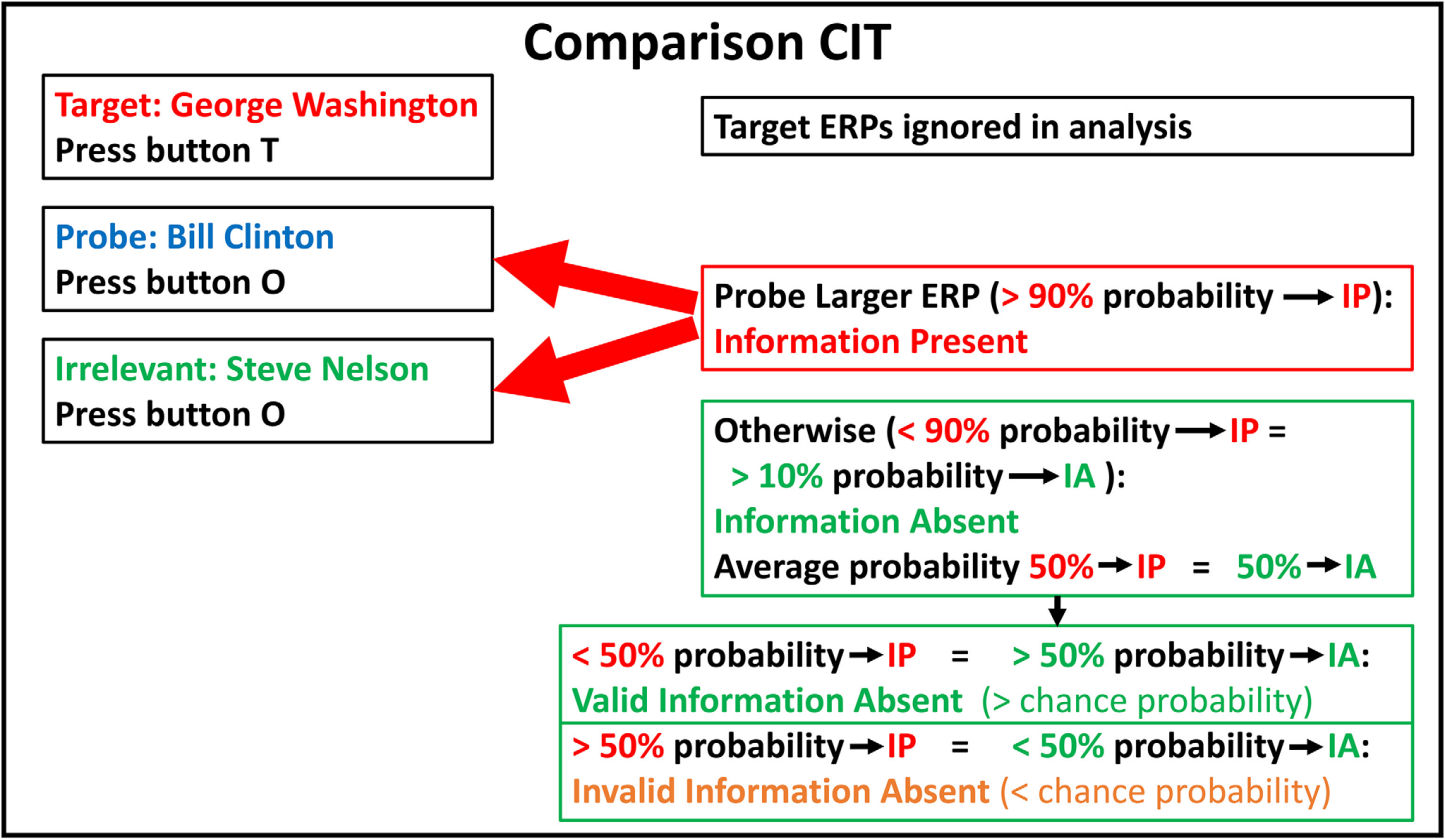
**Comparison CIT: stimuli, responses, and determinations**. Example of a comparison CIT to determine whether an individual has information about US political history. Subject instructions and button presses are the same as in Figure 1. IP, Information Present; IA, Information Absent.

Correct information-absent determinations are of two types, valid and invalid. Valid determinations are those that have a greater than 50% (chance) statistical confidence, i.e., a greater than chance computed probability of being correct. An invalid determination is a (correct) determination where the statistical confidence is less than chance (50%); that is, the computed probability that the determination is correct is less than 50%. Such a result is invalid because clearly one cannot validly report that “Our statistical procedure determined that the individual is information absent; the statistics computed a probability of [15%] that the determination is correct.” (This also applies to any other percentage lower than 50%.) Such a statement is not statistically meaningful or logically tenable. To be valid, the computed statistical confidence for a result must at least be better than chance (see Figure 2 and Appendix 2). To be scientifically meaningful and practically useful, it must be considerably better than chance.

There are serious scientific, mathematical, logical, and statistical flaws with the ERP-based comparison-CIT data-analysis procedure, as described in Farwell (2012, 2014), Farwell et al. (2013), and Appendix 2. These flaws cannot be corrected by simply changing the criterion for information present/absent determinations. We have implemented this procedure, however, because this is the way that the bootstrapping statistical confidence has been computed in all or virtually all of the comparison-CIT studies that have previously applied bootstrapping (e.g., Rosenfeld et al., 2008).

Figure 2 illustrates example stimuli, ERP responses, bootstrapping probabilities, and determinations for a hypothetical comparison CIT to determine if an individual has information regarding US political history.

## Results

**Figure 3:**
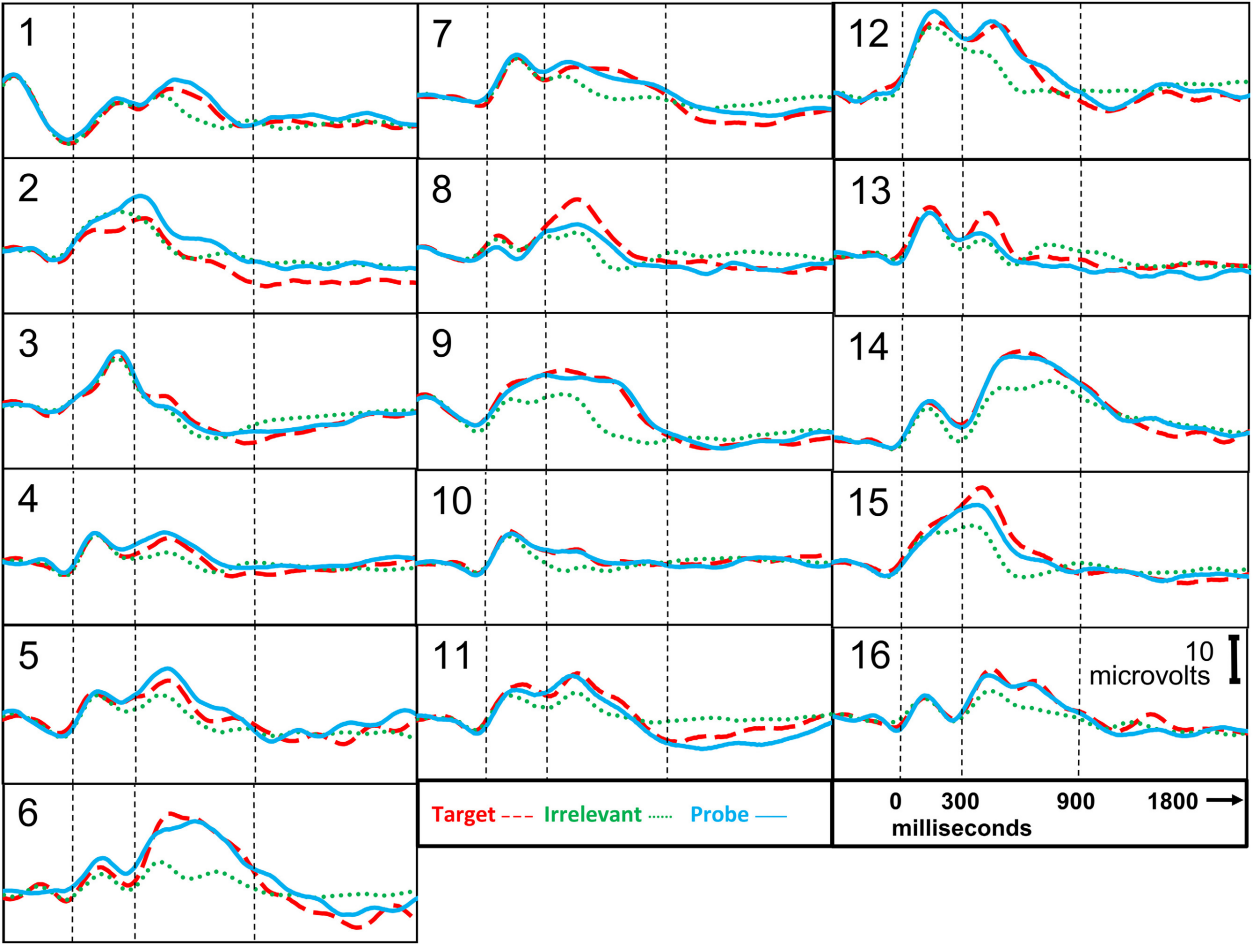
**ERP responses, information-present subjects**. Stimulus onset is at 0 ms. P300 epoch is 300–900 ms. P300 + LNP epoch is 300–1800 ms.

**Figure 4:**
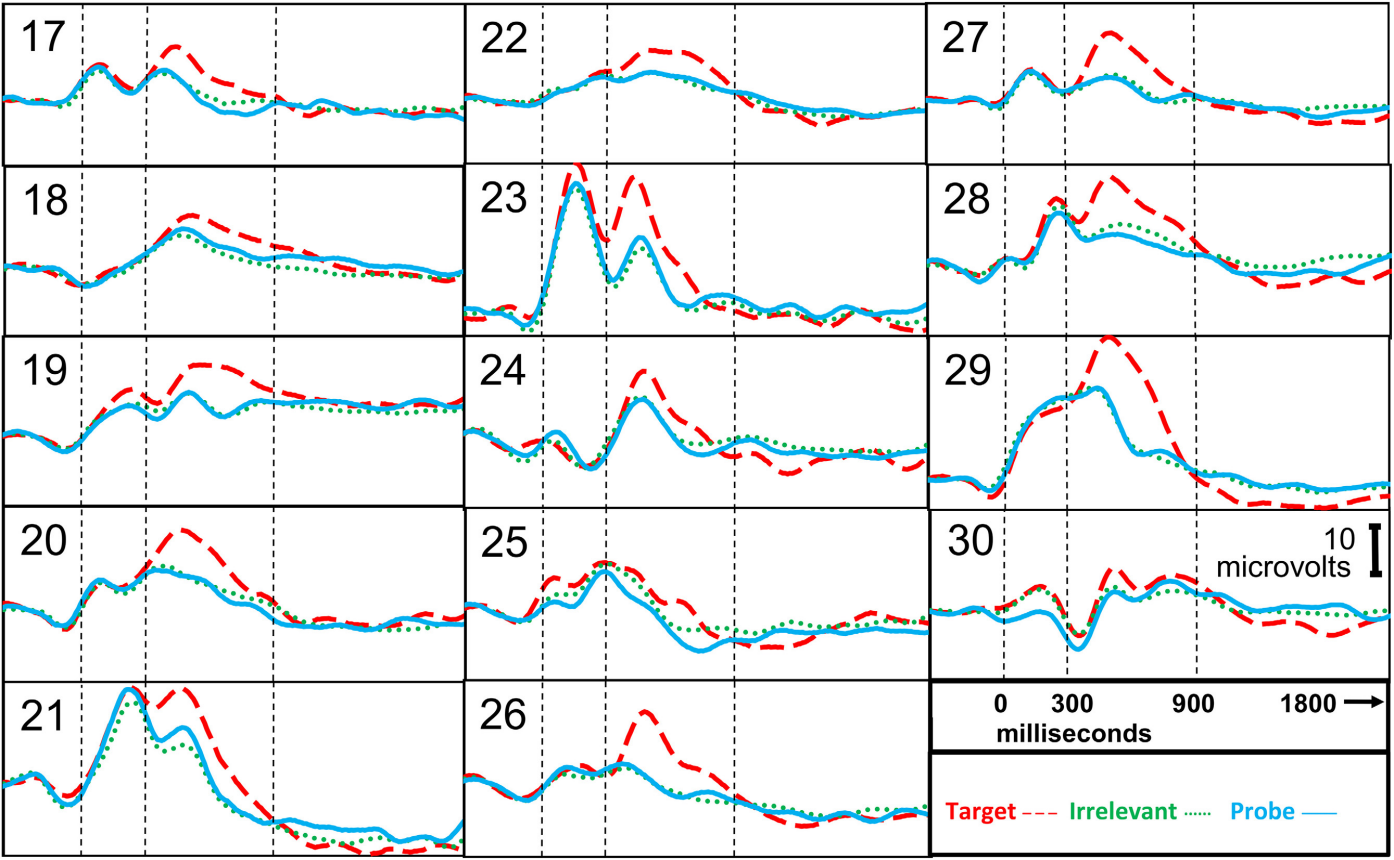
**ERP responses, information-absent subjects**. Stimulus onset is at 0 ms. P300 epoch is 300–900 ms. P300 + LNP epoch is 300–1800 ms.

The results are delineated in Tables 1–**4** and illustrated in Figures 3 and 4. Table 1 presents the error rate/accuracy of the results of the classification CIT, for both P300 + LNP and P300 analysis methods. Both P300 + LNP and P300 analysis methods produced 0% error rate, 100% accuracy. Both also produced no indeterminates.

**Table 1:** **Classification CIT error rate/accuracy of determinations with P300 + LNP and P300**.

**Classification CIT: error rate/accuracy with P300 + LNP and P300**			
Information-present subjects	Tests	16	100%
	Correct positives	16	100%
	False negatives	0	0%
	Indeterminates	0	0%
Information-absent subjects	Tests	14	100%
	Correct negatives	14	100%
	False positives	0	0%
	Indeterminates	0	0%
All subjects	Tests	30	100%
	Correct determinations	30	100%
	Errors	0	0%
	Indeterminates	0	0%
	Accuracy	30/30	100%
	Error rate	0/30	0%

Table 2 presents the error rate/accuracy of the comparison CIT. (Only correct determinations and errors are tabulated: the comparison CIT does not have an indeterminate category).

**Table 2:** **Comparison CIT error rate/accuracy**.

**Comparison CIT: error rate/accuracy**			
Information-present subjects	Tests	16	100%
	Correct positives	13	81%
	False negatives	3	19%
Information-absent subjects	Tests	14	100%
	Correct negatives	11	79%
	False positives	3	21%
All subjects	Tests	30	100%
	Correct determinations	24	80%
	Errors	6	20%
	Accuracy	24/30	80%
	Error rate	6/30	20%

Table 3 presents the individual determinations and the statistical confidences for each subject whose true state was information present. It compares the results obtained with the classification CIT with P300 + LNP with the other two methods: classification CIT with P300 and comparison CIT.

**Table 3:**
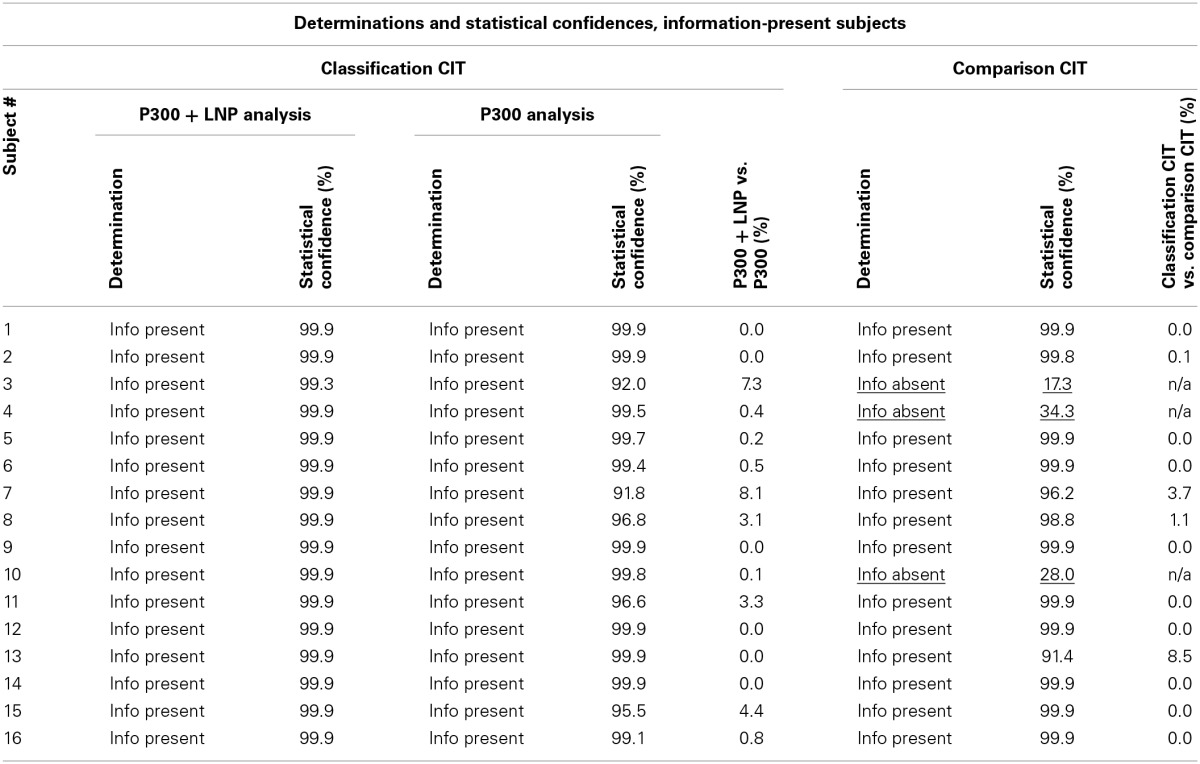
**Determinations and statistical confidences for information-present subjects**.

*Errors are underlined*.

Figure 3 presents the brain responses to probe, target, and irrelevant stimuli for each of the information-present subjects, averaged across all trials for each subject.

Table 4 presents the individual determinations and the statistical confidences for each subject whose true state was information absent. It compares the results obtained with the classification CIT with P300 + LNP with the other two methods: classification CIT with P300 and comparison CIT.

**Table 4:**
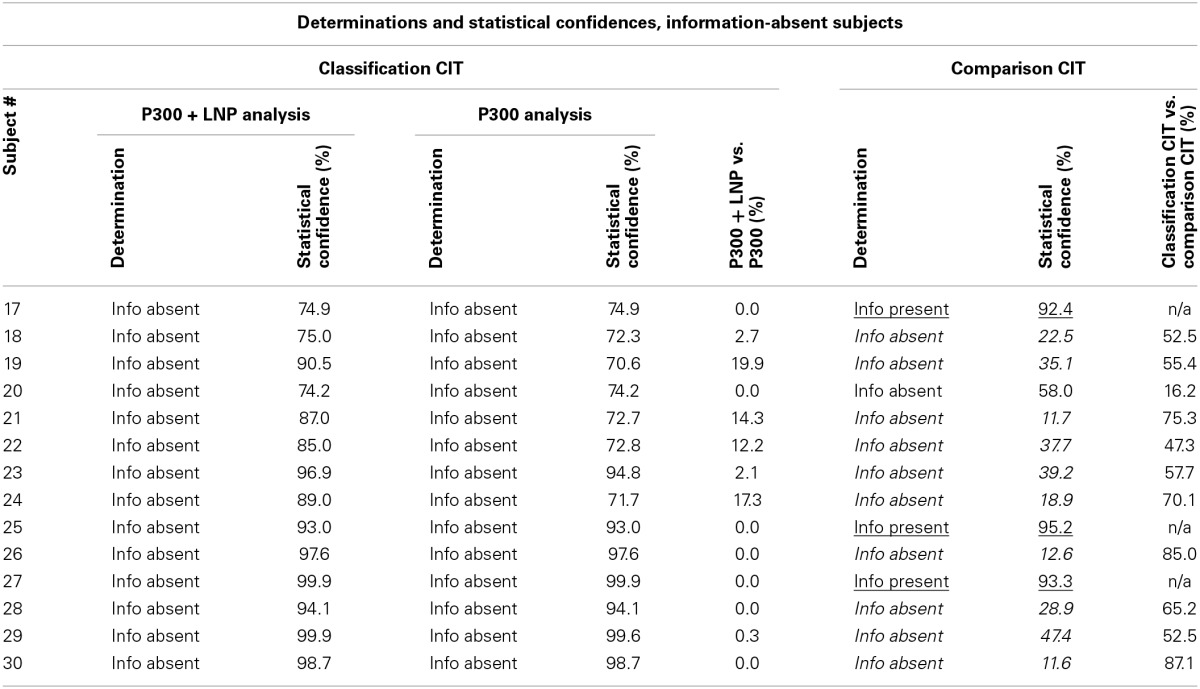
**Determinations and statistical confidences for information-absent subjects**.

*Errors are underlined; invalid determinations, i.e., correct determinations made with less than 50% (chance) statistical confidence, are in italics*.

Figure 4 presents the brain responses to probe, target, and irrelevant stimuli for each of the information-absent subjects, averaged across all trials for each subject.

### Results of the classification CIT with P300 + LNP analysis

All classification-CIT determinations with the P300 + LNP analysis were correct. Error rate was 0%: there were no false positives and no false negatives. Accuracy was 100%. Also, there were no indeterminates. Grier's A' (Grier, 1971) was 1.0.

All information-present statistical confidences were above the *a priori* criterion of 90%. All information-absent determinations were above the *a priori* criterion of 70% (in the opposite direction). Median statistical confidence was 99.9% with the P300 + LNP. Mean statistical confidence was 95.1% with the P300 + LNP.

All of the information-present determinations were made with a statistical confidence of at least 99%, and all but one were made with a statistical confidence of 99.9%. Median statistical confidence for information-present determinations was 99.9%, and mean statistical confidence was also 99.9%.

All information-present determinations exceeded the *a priori* criterion of 90% statistical confidence by at least 9 percentage points in the bootstrap probability computation. No information-present determinations were close to an indeterminate outcome. All information-present determinations were extremely far from a false negative. The lowest information-present determination was separated by a buffer of 69 percentage points in the bootstrap probability computation from the criterion for a false negative. (Exceeding the 70% probability for an information-absent determination would result in a false negative. This is equivalent to 100 – 70% = 30% probability for an information-present determination. Lowest information-present probability obtained was 99.3%, and 99.3 – 30% = 69.3%).

All of the information-absent determinations also exceeded the corresponding *a priori* criterion of 70% statistical confidence for information-absent determinations. Median information-absent statistical confidence with the P300 + LNP was 91.8%. Mean information-absent statistical confidence with the P300 + LNP was 89.7%.

All information-absent determinations were far from a false positive. The least statistically confident information-absent determination was separated by a buffer of 64 percentage points in the bootstrap probability computation from the criterion for a false positive. (Exceeding the 90% probability for an information-present determination would result in a false positive. This is equivalent to 100 – 90% = 10% probability for an information-absent determination. Lowest information-absent probability obtained is 74.2%, and 74.2 – 10% = 64.2%).

Statistical confidences for information-absent determinations were lower than for information-present determinations, however, and some were close to an indeterminate outcome. 9 information-absent determinations had statistical confidences of less than 95%. 6 had statistical confidences of less than 90%. 2 statistical confidences were less than 75% and were within 5 percentage points of an indeterminate outcome. Possible reasons for this are discussed below.

### Results of the classification CIT with P300 analysis

As with the classification-CIT P300 + LNP-based analysis, all determinations with the classification-CIT P300-based analysis were correct. Error rate was 0%: there were no false positives and no false negatives. Accuracy was 100%. Also, there were no indeterminates. Grier's A' (Grier, 1971) was 1.0. All information-present statistical confidences were above the *a priori* criterion of 90%. All information-absent determinations were above the *a priori* criterion of 70% (in the opposite direction). Median statistical confidence was 97.2% with the P300 alone. Mean statistical confidence was 91.9% with the P300 alone. For information-present subjects, median statistical confidence was 99.6%, and mean statistical confidence was 98.1%. For information-absent subjects, median statistical confidence was 84.0%, and mean statistical confidence was 84.8%. With the P300 analysis, 12 subjects (2 information present and 10 information absent) had statistical confidences of less than 95%, and 7 (all information absent) had statistical confidences of less than 75% and were within 5 percentage points of an indeterminate outcome. All determinations were very far from a false negative or false positive error.

### Comparing classification-CIT P300 + LNP analysis vs. P300 analysis

The classification-CIT P300 + LNP-based analysis produced significantly higher statistical confidences for individual determinations than the classification-CIT P300-based analysis (*p* < 0.0001, Wilcoxon matched-pairs signed rank test). The statistical confidence for the P300 + LNP-based analysis was an average of 3.2% higher than the statistical confidence for the P300-based analysis. In every case where there was a difference, the statistical confidence produced by the P300 + LNP was higher than that produced by the P300 alone. The P300 yielded a greater number of determinations with relatively low statistical confidence, close to an indeterminate outcome, than the P300 + LNP.

### Results of the comparison CIT

Error rate with the comparison CIT was 20% overall, 19% false negatives for information-present subjects and 21% false positives for information-absent subjects. (The comparison CIT does not have an indeterminate category.) Mean statistical confidence for correct determinations was 67.0%. The lowest statistical confidence for a correct determination was 11.6%. Median statistical confidence for correct determinations was 93.8%. For information-present subjects, statistical confidences for correct determinations were all over 90%, as required by the criterion of 90% probability for information-present determinations; median was 99.9%; mean was 98.9%. As predicted by the statistical model, statistical confidences for correct information-absent determinations were on average not better than chance (50%). Median was 28.9%; mean was 29.4%. Most of the correct information-absent determinations were invalid, i.e., made with less than a 50% (chance) statistical confidence.

### Classification CIT (P300 + LNP analysis) vs. comparison CIT

Even if we conservatively consider the 0% error rate of the classification CIT to be “less than 1%” for the sake of avoiding the anomalies of 0%, the comparison CIT produced more than an order of magnitude higher error rate than the classification CIT. This difference was significant (*p* < 0.05, sign test). Moreover, the comparison CIT produced significantly lower statistical confidences for correct determinations than the classification CIT (*p* < 0.0007, Wilcoxon matched-pairs signed rank test). On average, the comparison CIT produced statistical confidences 28.2 percentage points lower than those of the classification CIT in the bootstrap probability computation (for correct determinations). As predicted by the statistical model, this difference was particularly striking for information-absent subjects: comparison-CIT statistical confidences averaged 60.4 percentage points lower than classification-CIT statistical confidences for information-absent subjects. Correct statistical confidences for information-absent subjects with the comparison CIT averaged 29.4%, which is less than chance (50%).

## Discussion

### Conclusions

Our results suggest the following:

1. The classification CIT applying the brain fingerprinting methods and scientific standards described herein produces (a) significantly lower error rates (less than 1%; actually 0%) and (b) significantly higher statistical confidences than the comparison CIT.
2. Classification-CIT data analysis with the P300 plus the LNP produces significantly higher statistical confidences than analysis with the P300 alone. Error rates were less than 1% (actually 0%) for both methods.

Our results suggest that the classification CIT, when practiced according the methods and standards described here, is a reliable and valid method for detecting concealed information obtained in the course of real life that is characteristic of individuals with specific training, expertise, and/or affiliation with a particular agency or organization.

In our view, the minimum criteria for valid, reliable, and ethical field use for an ERP-based CIT are an error rate of less than 1% along with median statistical confidences of greater than 95%, including greater than 90% for both information-present and information-absent determinations. Our results, taken together with the results of our previous research and independent replications by others (see Farwell, 2012, 2014), suggest that the classification-CIT methods reported herein provide sufficient conditions to meet these criteria in the laboratory and the field. In our view, the methods applied in this research are sufficiently valid and reliable to be ethically applied in field use with substantial consequences to the outcome. These methods can be (and have been) reliably and effectively applied in field criminal cases.

Our results and those of all previous studies taken together (see Farwell, 2012, 2014) suggest that the comparison CIT with ERPs falls far short of these performance criteria for both error rate and statistical confidence. They also suggest that including the P300 + LNP in data analysis provides higher statistical confidences than P300 alone, but it is not a necessary condition for low error rate and high statistical confidences.

The most striking feature of the data reported to date, including the data of this study, is that there is a sharp bimodal distribution of error rates and statistical confidences, based on the following. One set of methods, as described here, applies the classification CIT and always has produced less than 1% error rate and greater than 95% median statistical confidences. Alternative methods, exemplified by Rosenfeld et al. (2008), Dietrich et al. (2014), and Meixner and Rosenfeld (2014), apply the comparison CIT and have produced an order of magnitude higher error rates, as well as statistical confidences averaging no better than 50% (chance) for information-absent determinations. Two reviews including all previous publications in English, Farwell (2012, 2014), documented that only the specific methods that substantially incorporate the 20 brain fingerprinting standards have so far reliably produced less than 1% error rate and greater than 95% median statistical confidences in the laboratory and the field. These are the methods applied in this research. The results of this research suggest that the differences in statistical methods between the classification CIT and the comparison CIT are responsible, at least in large measure, for the extremely large differences between the statistical confidences achieved empirically by the respective techniques.

Our experiment is a specific screening test where the information detected was relevant to expertise and experience in a particular field. Subjects obtained the tested information in the course of real life over a period of years, completely unconnected to any experimental procedures at the time the information was gained by the subjects. The results contribute to the accumulating evidence [e.g., the FBI and bomb-maker studies in Farwell et al. (2013)] that these methods provide a reliable and accurate technique for such applications.

In a few previous studies, real-life information has been detected for real-world crimes with life-changing consequences [the real crime study of Farwell et al. (2013)], and other real-life specific events [experiment 2 of Farwell and Donchin (1991), Farwell and Smith (2001), and people (Meijer et al., 2007)]. Almost all other ERP-based CIT studies have detected information obtained by the subjects in the course of a laboratory information-imparting procedure such as a mock crime (Farwell, 2012, 2014). Meixner and Rosenfeld (2014) conducted a comparison CIT in detecting information regarding unscripted activities that subjects had videotaped the previous day in conjunction with the experiment. Such activities are different from real-life activities; no one would commit an actual crime under such circumstances. Meixner and Rosenfeld failed to cite the previous peer-reviewed publications reporting field studies on real-world crimes and other real-life events, and falsely claimed to be the first study investigating information obtained in real life. Their results were similar to those of other comparison-CIT studies, including the results reported here (Appendix 2).

### Field applications in real-world crimes

These results complement the results of previous studies (Farwell and Smith, 2001; Farwell et al., 2013) in which the classification CIT was applied to detect concealed information regarding specific events, including field applications involving real-world major crimes. Field applications with life-changing or life-threatening consequences to the outcome involve more demanding conditions, including high motivation and other emotional factors, complexities, logistical challenges, uncontrolled context, and other factors that are difficult to bring under experimental control. We have conducted classification-CIT tests in real-world situations in which all of these demanding conditions were present, for example, tests on both innocent and guilty individuals who were facing the death penalty for murder as well as individuals who had already been convicted of murder and were attempting to establish their innocence. In such situations, low error rate and high statistical confidence are obviously of paramount importance.

The low error rate produced by the classification-CIT methods applied was one of the key features considered when brain fingerprinting was ruled admissible in court in the Harrington murder case (Harrington v. State, 2001; Farwell and Makeig, 2005; Roberts, 2007) in which a falsely convicted man was ultimately exonerated and freed. Extremely low error rates and high statistical confidences were equally important for using the ERP-based classification CIT to bring perpetrators such as serial killer J. B. Grinder to justice (Farwell, 2012; Farwell et al., 2013).

### What are the primary methods that may have contributed to the low error rate and high statistical confidences reported herein?

The following features of the methods practiced in this research may have contributed to the low error rate and high statistical confidences obtained here and in previous studies with these methods. The primary difference between this research and various studies that produced an order of magnitude higher error rates and average 50% (chance) statistical confidence for information-absent determinations is that we used the classification CIT, rather than the comparison CIT. The comparison CIT was used in virtually all of the studies that have reported high error rates and low statistical confidences (Farwell, 2012, 2014). We applied a classification statistical algorithm, rather than a comparison algorithm, in data analysis. We used each subject's response to situation-relevant target stimuli as a template for that subject's brain response to known, situation-relevant information. We used the subject's response to irrelevant stimuli as a template for that subject's brain response to unknown or irrelevant information. We then used bootstrapping to classify the subject's brain response to the probe stimuli as being more similar to his response to known information relevant to the investigated situation (targets) or to her response to unknown, irrelevant information (irrelevants). This allowed us to make both information-present and information-absent determinations with a high statistical confidence that the determination made is in fact correct in light of the effect size and variability in this subject's data, and that the opposite determination would be incorrect (see Appendix 2).

By contrast, the comparison CIT ignores the target responses and compares only the probe and irrelevant responses, resulting in lower accuracy and statistical confidences averaging 50% (chance) for information-absent determinations, as described in Appendix 2 and in Farwell (2012, 2014) and Farwell et al. (2013).

One previous error and resulting misrepresentation (we presume inadvertent) has caused considerable confusion in this regard (see Appendix 2). Rosenfeld et al. (2004) purported to be a replication of Farwell and Donchin (1991), but in fact did not use the two-tailed classification CIT of Farwell and Donchin, but rather a one-tailed method similar to the comparison CIT of Rosenfeld's other studies (see Appendix 2). The high error rates and low statistical confidences of Rosenfeld et al. (2004) have been mistakenly cited (Rosenfeld et al., 2008) as evidence that Farwell and Donchin's classification-CIT methods are inaccurate (and susceptible to countermeasures), whereas in fact those results only demonstrate that Rosenfeld et al.'s fundamentally different methods are inaccurate (and susceptible to countermeasures) (Farwell, 2011).

Our current results demonstrate once again that the comparison CIT produces higher error rates and lower statistical confidences than the classification CIT, even when the other brain fingerprinting scientific standards (Appendix 1) are substantially met.

### What methods are necessary to produce high statistical confidences with bootstrapping?

To produce high statistical confidences with bootstrapping, first of all the methods applied must be effective in producing the predicted experimental effects in the brain responses. Given that, what else is necessary in the statistical methods?

The statistical model of the classification CIT predicts high statistical confidences for both information-present and information-absent determinations, and this is what has been consistently reported. The statistical model of the comparison CIT predicts average statistical confidences no better than chance (50%) for information-absent determinations, and this also is what has been reported in the studies to date (Farwell, 2012, 2014; Appendix 2).

The bootstrapping technique applied here, and in all studies implementing the 20 standards, uses a classification CIT. It computes the probability that the probe responses are more similar to the target responses than to the irrelevant responses. 100% minus this is the probability that the probe responses are more similar to the irrelevant responses. This allows for a result of a high statistical confidence for both information-present and information-absent determinations. The comparison CIT computes the probability that the probe responses are larger than the irrelevant responses. This probability is expected to be high for information-present subjects. For information-absent subjects, probe and irrelevant responses are expected to be identical, so the expected value of the probability that the probe response is larger is 50%. This is the expected bootstrap probability that information present is the correct determination, the expected information-present statistical confidence. This makes the expected information-absent probability or statistical confidence also 50% (i.e., 100 – 50% = 50%). Thus, the expected statistical confidence for an information-absent determination with the comparison CIT is 50% (chance), assuming that the methods and statistics work as predicted. This is described in detail in Appendix 2.

Statistical confidences for information-absent determinations reported for the comparison CIT to date have in every study averaged approximately 50% (or less). Approximately half of the information-absent statistical confidences reported have been invalid, that is, less than 50% (chance) (Farwell, 2012, 2014). In approximately half of the cases, authors reported less than a 50% probability that the chosen (information-absent/“innocent”) determination was correct, according to the statistics used to arrive at the determination. For example, in Meixner et al. (2009, p. 215; Table 2; “innocent” subject 11) the subject was determined to be “innocent” (information absent) when the computed probability was 85% that “guilty” was the correct determination (i.e., that the probe P300 was larger than the irrelevant P300). Statistical confidence for this (correct) determination was 15%, far less than chance. 60% of subjects correctly determined to be “innocent” in this condition had statistical confidences of less than 50% (chance) that this determination was correct (i.e., had invalid results).

The comparison CIT in this research, as in previous comparison CIT studies (e.g., Rosenfeld et al., 2008; Dietrich et al., 2014; Meixner and Rosenfeld, 2014; see Appendix 2), produced markedly higher error rates and lower statistical confidences than those of the classification CIT. The results of this research, along with the results of all previous research (Farwell, 2012, 2014), suggest that applying the classification CIT rather than the comparison CIT is not only a sufficient condition, but is also a necessary condition for obtaining median 95% statistical confidences, and in particular for obtaining greater than 90% median statistical confidences for information-absent subjects—or even for obtaining greater than chance (50%) median statistical confidences for information-absent subjects (see Appendix 2).

### What additional methods may have contributed to the low error rate and high statistical confidences reported?

We used double-centered correlation as a measure of the similarity of the probe response to the target or irrelevant response (see Appendix 2). This metric has the advantage of including the entire response, not just a single point (or average of a few points) such as the peak amplitude or the difference between the positive P300 peak and the negative LNP peak. It inherently takes into account not only the peak amplitude, but also the latency and morphology of the full ERP. With the correlation metric, latency differences between probe, target, and irrelevant responses, as well as individual differences in latency and morphology of the ERP, contribute to the characterization of the response and hence to the accuracy and statistical confidence of the result. The information contained in such differences is lost when the P300 is characterized by a single number such as peak amplitude, as applied in, for example, Rosenfeld et al. (2008) and Meixner and Rosenfeld (2014). Our more comprehensive characterization of the waveform may be one reason for the low error rate and high statistical confidence of this research and the previous studies that have used this method.

The term “brain fingerprinting” arises from an analogy to fingerprints that has several facets. Fingerprinting matches prints from the crime scene with prints on the fingers. DNA “fingerprinting” matches biological samples from the crime scene with biological samples from the suspect. “Brain fingerprinting” matches information from the crime scene with information stored in the brain of the subject. Moreover, fingerprints calculate a match based on multiple characteristics. In the autonomic skin conductance response (SCR) as well as in comparison-CIT P300 measurements, the response is generally defined in terms of a single parameter. With SCR this may be the maximum conductance increase that occurs following stimulus onset. With the P300 this is usually peak-to-post-(negative)-peak amplitude, defined as a single number. Brain fingerprinting, like fingerprinting, uses multiple facets of the response to compute a match between known patterns and the pattern tested, taking into account not only the peak amplitude but also the morphology and time course of both the positive and negative peaks in the response.

We used situation-relevant targets. Target stimuli, like probes, were relevant to the information detected. This makes the targets more similar to the probes for the subjects who possess the relevant information, and thus may increase accuracy and statistical confidence (Farwell et al., 2013). The difference between the targets and the probes was that the targets were identified to the subject in subject instructions and required a special button press, and probes were not identified in instructions and required the same button press as irrelevants.

### What are the possible shortcomings of the current study?

Despite the 0% error rate, the results of this research have certain shortcomings when considered in light of the rigorous requirements demanded by field applications with major consequences. Although all determinations were correct and very far from a false positive or false negative error, the statistical confidence of some determinations was low enough to be close to an indeterminate. This contrasts with previous studies (Farwell and Smith, 2001; Farwell et al., 2013), where all determinations were correct and also far from an indeterminate result.

One reason for this shortcoming may be the relatively low number of trials presented in this research, and consequently a lower signal-to-noise ratio. [This does not, however, explain why the FBI agent study (Farwell et al., 2013) produced higher statistical confidences than this research, without more trials. Further research may identify other differentiating factors]. This research used only 84 probe trials and 84 target trials in the averages. In previous studies where we have used at least 100 probe trials and an equal number of targets, statistical confidences have been considerably higher. Moreover, the results of these two studies demonstrate that while brain fingerprinting standard 13 (use at least 100 probe trials—see Appendix 1) has been shown to be useful for producing optimal results, it is not absolutely requisite for achieving high levels of accuracy or statistical confidence. In other words, standard 13 is part of the well-established set of sufficient conditions, but is not a necessary condition for low error rate and high statistical confidences.

### Summary

We used the classification CIT to detect information gained by subjects in the course of real life. They gained the tested information in real-life events over a period of years before, and completely unrelated to, the experimental procedures. This was a specific screening or focused screening test, rather than a specific issue test. That is, rather than detecting information obtained at a particular place and time (such as while committing a crime), we detected information known to people with specific training, expertise, and organizational affiliation, specifically knowledge of military medicine by US Navy military medical experts. Subjects obtained this knowledge through a variety of experiences at different times and places for different individuals.

In detecting this concealed information, the classification CIT with the P300 + LNP produced 0% error rate and median 99.9% statistical confidence for individual determinations, a significantly lower error rate and higher statistical confidences than those produced by the comparison CIT.

Although the classification-CIT methods using both the P300 and the P300 + LNP produced the same 0% error rate, the P300 + LNP produced significantly higher statistical confidences for individual determinations. In continued field use, with the concomitant demanding conditions, eventually errors (or at least indeterminates) may occur with these methods. If so, then the higher statistical confidences produced by the P300 + LNP (rather than the P300 alone) can be expected to result in lower error rates when the error rate is non-zero.

In our view, to reliably produce the predicted experimental effect and to be viable for field use, a technique must consistently produce less than 1% error rate, along with high statistical confidences for both information-present and information-absent determinations.

The results of this study, together with the results of similar studies such as the FBI agent study and the bomb-maker study of Farwell et al. (2013), suggest that the classification CIT methods specified here, when the full P300 + LNP epoch is employed in data analysis, can be used effectively in specific screening tests to detect knowledge characteristic of individuals with specific training, expertise, and/or affiliation with a particular agency or organization. In our current study, this was accomplished in a specific screening test under controlled conditions, with the limitations inherent thereto. Prior research has applied these same methods in field conditions in a specific issue test in investigating actual crimes, with the concomitant complications related to motivation, emotions, logistics, experimental control, and other uncontrollable factors. Taken together with previous successful field applications in real-world criminal investigations, our results suggest that these methods may have application in both national security and law enforcement, for instance in identifying trained terrorists, bomb makers, members of a terrorist cell, hostile intelligence agents, members of an organized crime organization, and others with specific knowledge, expertise, training, and/or affiliations of interest.

## Conflict of interest statement

Research contract 92-F138600-000 US Central Intelligence Agency (CIA). Richardson was an FBI agent at time of research. US Navy and USUHS provided facilities and subjects. Farwell is inventor in US patents (#7,689,272; 5,363,858; 5,406,956; 5,467,777) and one UK patent (# GB2421329) relevant to the research. Farwell is the Chairman and Chief Scientist of Brain Fingerprinting Laboratories, Inc., member of Brain Fingerprinting, LLC and Brainwave Science, LLC, commercial neuroscience companies.

## Footnotes

^1^To obtain statistical confidences for each individual determination, Farwell, Donchin, Wasserman, and Bockenholt collaborated to introduce the statistical technique of bootstrapping in the field of psychophysiology. This first was published in conference abstracts by Farwell and Donchin (1986, 1988a), and full papers by Wasserman and Bockenholt (1989) and Farwell and Donchin (1991), which reported the same studies as the abstracts. Wasserman and Bockenholt used Farwell and Donchin's application of bootstrapping in the classification CIT as an example of the correct use of the technique.

^2^The study reported here appeared first as a conference abstract, Farwell and Richardson (2006).

## Appendix 1

### Brain fingerprinting scientific standards

1. Use equipment and methods for stimulus presentation, data acquisition, and data recording that are within the standards for the field of cognitive psychophysiology and event-related brain potential research. These standards are well documented elsewhere. For example, the standard procedures Farwell introduced as evidence in the Harrington case were accepted by the court, the scientific journals, and the other expert witnesses in the case (Harrington v. State, 2001; Farwell, 2012, 2014). Use a recording epoch other expert witnesses in the case. Use a recording epoch long enough to include the full P300 + LNP (i.e., P300-MERMER). For pictorial stimuli or realistic word stimuli, use at least a 1,800-millisecond recording epoch. (Shorter epochs may be appropriate for very simple stimuli).
2. Use correct electrode placement. The P300 and P300-MERMER are universally known to be maximal at the midline parietal scalp site, Pz in the standard International 10–20 system.
3. Apply brain fingerprinting tests only when there is sufficient information that is known only to the perpetrator and investigators. Use a minimum of six probes and six targets.
4. Use stimuli that isolate the critical variable: the subject's knowledge or lack of knowledge of the probe stimuli as significant in the context of the investigated situation. Obtain the relevant knowledge from the criminal investigator (or for laboratory studies from the knowledge-imparting procedure such as a mock crime and/or subject training session). Divide the relevant knowledge into probe stimuli and target stimuli. Probe stimuli constitute information that has not been revealed to the subject. Target stimuli contain information that has been revealed to the subject after the crime or investigated situation.
5. If initially there are fewer targets than probes, create more targets. Ideally, this is done by seeking additional known information from the criminal investigators. Note that targets may contain information that has been publicly disclosed. Alternatively, some potential probe stimuli can be used as targets by disclosing to the subject the specific items and their significance in the context of the investigated situation.
6. For each probe and each target, fabricate several stimuli of the same type that are unrelated to the investigated situation. These become the irrelevant stimuli. Use stimuli that isolate the critical variable. For irrelevant stimuli, select items that would be equally plausible for an information-absent subject. The stimulus ratio is approximately one-sixth probes, one-sixth targets, and two-thirds irrelevants.
7. Ascertain that the probes contain information that the subject has no known way of knowing, other than participation in the investigated situation. This information is provided by the criminal investigator for field studies, and results from proper information control in laboratory studies.
8. Make certain that the subject understands the significance of the probes, and ascertain that the probes constitute only information that the subject denies knowing, as follows. Describe the significance of each probe to the subject. Show him the probe and the corresponding irrelevants, without revealing which is the probe. Ask the subject if he knows (for any non-situation-related reason) which stimulus in each group is situation-relevant/crime-relevant. Describe the significance of the probes and targets that will appear in each test block immediately before the block.
9. If a subject has knowledge of any probes for a reason unrelated to the investigated situation, eliminate these from the stimulus set. This provides the subject with an opportunity to disclose any knowledge of the probes that he may have for any innocent reason previously unknown to the scientist. This will prevent any non-incriminating knowledge from being included in the test.
10. Ascertain that the subject knows the targets and their significance in the context of the investigated situation. Show him a list of the targets. Describe the significance of each target to the subject.
11. Require an overt behavioral task that requires the subject to recognize and process every stimulus, specifically including the probe stimuli, and to prove behaviorally that he has done so on every trial. Detect the resulting brain responses. Do not depend on detecting brain responses to assigned tasks that the subject can covertly avoid doing while performing the necessary overt responses.
12. Instruct the subjects to press one button in response to targets, and another button in response to all other stimuli. Do not instruct the subjects to “lie” or “tell the truth” in response to stimuli. Do not assign different behavioral responses or mental tasks for probe and irrelevant stimuli.
13. In order to obtain statistically robust results for each individual case, present a sufficient number of trials of each type to obtain adequate signal-to-noise enhancement through signal averaging. Use robust signal-processing and noise-reduction techniques, including appropriate digital filters and artifact-detection algorithms. The number of trials required will vary depending on the complexity of the stimuli, and is generally more for a field case. In their seminal study, Farwell and Donchin (1991) used 144 probe trials. In the Harrington field case, a murder case wherein brain fingerprinting and Farwell's testimony in it were admitted as scientific evidence, Farwell used 288 probe trials (Harrington v. State, 2001; Farwell et al., 2013). In any case, use at least 100 probe trials and an equal number of targets. Present three to six unique probes in each block.
14. Use appropriate mathematical and statistical procedures to analyze the data. Do not classify the responses according to subjective judgments. Use statistical procedures properly and reasonably. At a minimum, do not determine subjects to be in a category where the statistics applied show that the determination is more likely than not to be incorrect, i.e., statistical confidence is less than 50%.
15. (a) Use a mathematical classification algorithm, such as bootstrapping on correlations, that isolates the critical variable by classifying the responses to the probe stimuli as being either more similar to the target responses or to the irrelevant responses. (b) In a forensic setting, conduct two analyses: one using only the P300 (to be more certain of meeting the standard of general acceptance in the scientific community), and one using the P300 + LNP (P300-MERMER) to provide the current state of the art.
16. Use a mathematical data-analysis algorithm that takes into account the variability across single trials, such as bootstrapping.
17. Set a specific, reasonable statistical criterion for an information-present determination and a separate, specific, reasonable statistical criterion for an information-absent determination. Classify results that do not meet either criterion as indeterminate. Recognize that an indeterminate outcome is not an error, neither a false positive nor a false negative. Error rate is the percentage of information-present or information-absent determinations that are false positives and false negatives respectively; accuracy is 100% minus the error rate.
18. Restrict scientific conclusions to a determination as to whether or not a subject has the specific situation-relevant knowledge embodied in the probes stored in his brain. Recognize that brain fingerprinting detects only presence or absence of information – not guilt, honesty, lying, deception, or any action or non-action. Do not offer scientific opinions on whether the subject is lying or whether he committed a crime or other act. Recognize that the question of guilt or innocence is a legal determination to be made by a judge and jury, not a scientific determination to be made by a scientist or a computer.
19. Evaluate error rate/accuracy based on actual ground truth. Ground truth is the true state of what a scientific test seeks to detect. Brain fingerprinting is a method to detect information stored in a subject's brain. Ground truth is whether the specific information tested is in fact stored in the subject's brain. Establish ground truth with certainty through post-test interviews in laboratory experiments and in field experiments wherein subjects are cooperative. Establish ground truth insofar as possible through secondary means in real-life forensic applications with uncooperative subjects. Recognize that ground truth is the true state of what the subject in fact knows, not what the experimenter thinks the subject should know, not what the subject has done or not done, and not whether the subject is guilty, or deceptive.
20. Make scientific determinations based on brain responses. Do not attempt to make scientific determinations based on overt behavior that can be manipulated, such as reaction time.

## Appendix 2

### Previous research on sufficient and necessary conditions for criterion low error rates and high statistical confidences

#### Summary of previous results

The most striking feature of the data reported to date, including this research, is the sharp bimodal distribution of error rates and statistical confidences. So far, the only proposed explanation for this bimodal distribution is that a specific set of classification-CIT methods, as described here and in the 20 brain fingerprinting standards (Appendix 1), have consistently produced less than 1% error rate and greater than 95% median statistical confidences for individual determinations (Farwell, 2012, 2014; Farwell et al., 2013). Alternative methods, exemplified by Rosenfeld et al. (2004, 2008), Dietrich et al. (2014), and Meixner and Rosenfeld (2014), have produced an order of magnitude higher error rates, as well as statistical confidences averaging no better than chance (50%) for information-absent (“innocent”/“nonknowledgeable”) determinations. By applying two different data-analysis methods to the same data, our research directly addresses a fundamental difference in methods: the classification CIT vs. comparison CIT. This fundamental difference in methods accounts for a major difference in the results reported by previous studies applying the respective methods.

Previous results can be summarized as follows. All data are consistent with the hypothesis that standards 1–20 (Appendix 1) provide sufficient conditions for an ERP-based CIT to obtain less than 1% error rate and greater than 95% median statistical confidence, including greater than 90% statistical confidence for both information-present and information-absent determinations. At least some of these standards constitute necessary conditions. Several previous studies (e.g., Johnson and Rosenfeld, 1992; Rosenfeld et al., 2008; Dietrich et al., 2014; Meixner and Rosenfeld, 2014) demonstrated that applying comparison-CIT methods that fail to meet Standards 3–6, 8–15, and 17–20 resulted in high error rates, low statistical confidences, and invalid results. For reviews of all relevant studies in English to date, including a detailed discussion of which of the 20 standards were found to be necessary conditions in which studies, see Farwell (2012, 2014). Among the necessary conditions are standards 14, 15a, and 17, which describe the classification CIT and distinguish it from the comparison CIT. Standard 11, which requires subjects to read and process each stimulus and prove with a differential button press that they have done so, is a necessary condition for tests on motivated subjects in field conditions with major consequences to the outcome, but not for tests with accommodating subjects in the absence of non-trivial consequences. Standard 13, which requires a minimum number of probe trials in the average, is not a necessary condition, but does contribute to higher statistical confidences and potentially to higher accuracy.

#### Why the classification CIT (standards 14, 15a, and 17) is a necessary condition for high statistical confidences

In the classification CIT, probes and targets are both relevant details about the investigated situation. For information-present subjects, both are expected to result in essentially the same ERP, containing a large P300 and LNP. For an information-absent subject, the irrelevants and (unrecognized) probes are indistinguishable and equally irrelevant, and are expected to elicit identical ERPs lacking a large P300 and LNP. Classification-CIT bootstrapping asks the statistical question, “What is the probability that the probes are more similar to the targets than to the irrelevants?” 100% minus this is the probability that the probes are more similar to the irrelevants. The expected value of this statistic is 100% in the information-present direction (probes resemble targets) for information-present subjects, and 100% in the information-absent direction (0% in the information-present direction; probes resemble irrelevants) for information-absent subjects (see Figure 1). Thus, if the data are as predicted, the classification CIT provides a high statistical confidence for both information-present and information-absent determinations.

The comparison CIT ignores targets and asks the statistical question, “What is the probability that the probe ERPs are larger than the irrelevant ERPs?” For an information-present subject, the expected value is 100%. If the ERPs are as predicted, this method can deliver a high statistical confidence for information present subjects. That is, it can provide a high probability that information present is the correct determination. If the bootstrap probability that information present is correct is over 90%, the subject is determined to be information present (see Figure 2). If the bootstrap probability that information present is correct is less than 90%—equivalent to a probability of greater than 10% (i.e., 100–90%) that information absent is correct—the subject is determined to be information absent. Subjects are determined to be information absent with as low as a 10.1% statistically computed probability that this determination is correct. For an information-absent subject, probe and irrelevant ERPs are expected to be identical, so the expected value of the probability that probes are larger is 50%. This is the expected information-present statistical confidence, which makes the expected information-absent statistical confidence also 50% (i.e., 100% – 50% = 50%). This means that if the ERPs are as predicted and the statistics work as designed, the average statistical confidence for information-absent determinations will be 50% (chance). In all comparison-CIT studies reported to date (Farwell, 2012, 2014), the average statistical confidence for information-absent subjects has been approximately 50% (or less), and subjects have been determined to be information absent with as low as 10.1% statistical confidence or computed probability that this determination is correct. In summary, the comparison CIT, in accord with the predictions of the statistical model, has in every study to date produced statistical confidences averaging no better than chance for information-absent determinations. Thus, applying the classification CIT rather than the comparison CIT is a necessary condition for obtaining high statistical confidences, or even better-than-chance statistical confidences for information-absent subjects^3^.

#### Corrections regarding previous studies

Two major errors have led to considerable confusion and misinformation in the literature. Johnson and Rosenfeld (1992) cited Farwell and Donchin (1988a, 1991) and Wasserman and Bockenholt (1989) as the origin of the bootstrapping method, but they did not apply the classification-CIT method introduced by Farwell, Donchin, Wasserman, and Bokenholt. They applied a comparison CIT. Several other subsequent studies by the same group and others have cited Farwell and Donchin and Wasserman and Bockenholt as the source of their bootstrapping method, but have applied the comparison CIT instead. This has led to confusion and misinformation because the results of these two methods are substantially different. In particular, the high error rates and low statistical confidences characteristic of the comparison CIT have sometimes been falsely attributed to the classification CIT (e.g., Rosenfeld et al., 2004, 2008) or to all ERP-based CITs (see Farwell, 2012, 2014; Farwell and Richardson, 2013). Our data demonstrate that these two methods are substantially different not only in experimental design but also in results, for both error rate and statistical confidence.

Another error and consequent (good faith) misrepresentation has led to some apparently (but not actually) highly anomalous results in the literature. Rosenfeld et al. (2004) reported the high error rates and low statistical confidences that are typical of comparison-CIT studies that did not implement the 20 brain fingerprinting scientific standards, but mistakenly characterized their study as a replication of Farwell and Donchin (1991), the original classification-CIT study substantially implementing the standards. The error rate in the “FIT” condition, which was mistakenly described as a replication of Farwell and Donchin, was 46% in one group and 31% in another (without countermeasures), and even higher with countermeasures. Both of these are obviously higher than the less-than-1% error rate that has been achieved (both with and without countermeasures) in all instances in which the Farwell and Donchin methods were actually applied. The reason for this discrepancy is that, although Rosenfeld et al. characterized their methods as a replication of Farwell and Donchin, the actual methods they applied, as in that group's other studies, did not implement over half of the 20 brain fingerprinting standards, specifically standards 3–6, 8–10, 12–15, and 17–20 (Farwell, 2012, 2014; Farwell and Richardson, 2013). Among many other fundamental differences, they did not use the classification-CIT bootstrapping method of Farwell and Donchin and Wasserman and Bockenholt (1989). Thus, the high error rates Rosenfeld et al. reported are consistent with the high error rates of the other studies that did not implement many of the same standards. Rosenfeld et al. erroneously concluded that their results showed that the Farwell and Donchin method was inaccurate and susceptible to countermeasures (Rosenfeld et al., 2008), whereas in fact their results showed that their fundamentally different method is inaccurate and susceptible to countermeasures.

When the distinction between the classification CIT and the comparison CIT, and the other standards, are taken into account, the pattern of results in the literature is clear. Two different sets of methods produce two different sets of results. One set of methods, the classification CIT implementing the 20 standards, always has produced low error rates and high statistical confidences. Another, different set of methods implementing the comparison CIT has produced high error rates and low statistical confidences. In light of this distinction, the bimodal distribution of error rates and statistical confidences is explicable and even predictable. When this fundamental distinction is ignored or blurred, the literature inexplicably appears to contain two strikingly different groups of results for implementations of one undifferentiated method. Our experiment and results contribute to the clarification of this distinction in methods and the concomitant difference in results.

#### Why a required button-press discrimination for all stimuli (standard 11) is a necessary condition

The comparison CIT has been implemented in two different experimental designs for stimulus presentation and subject responses. One is the same as the three-stimulus target-probe-irrelevant design applied in the classification CIT and in our comparison-CIT experiment. The difference between the classification and comparison CITs with this design is in the data analysis: *classifying* the probe ERPs as being more similar to the target ERPs or the irrelevant ERPs (or neither—indeterminate) vs. ignoring the targets and *comparing* the probe ERPs with the irrelevant ERPs to determine whether the probe ERPs are significantly larger. Another version of the comparison CIT uses a four-stimulus “complex trial protocol” design (Rosenfeld et al., 2008). Each trial presents two stimuli. The first is always either a target or a non-target. The second is always either a probe or an irrelevant. Targets and non-targets are a completely different type of stimuli from probes and irrelevants, e.g., meaningless numbers (target: “six,” nontargets “one” through “five”). Thus, the targets do not provide a template for an information-present response, and without such a template the classification CIT cannot be used. The four-stimulus design must use the comparison-CIT data-analysis algorithm.

In the three-stimulus design, subjects are required to read and process every stimulus, decide if it is a target or not, and push the appropriate button. All subjects, regardless of motivation, are required to perform the same information-processing and button-press tasks. Subject strategies for responding to the four-stimulus complex trial protocol comparison-CIT design, by contrast, differ substantially depending on the motivation of the subject. Subjects are required to distinguish by a button press between targets and non-targets, so they must read and process them, regardless of motivation. After they have pushed the correct button in response to the target/nontarget, they know that no discrimination will be required in response to the next stimulus. They know for certain that either a probe or an irrelevant will appear, and they simply push the same button whatever appears. Accommodating laboratory subjects read and process the probe/irrelevant stimuli as instructed, push the button, and respond with different ERPs to probes and irrelevants, resulting in better-than-chance accuracy of the test (Meixner and Rosenfeld, 2014). Motivated subjects with life-changing consequences to the outcome recognize that they do not need to read and process the probe/irrelevant stimuli in order to push a button whenever something appears on the screen shortly after the target/nontarget discrimination and button press. Thus, motivated subjects do not read and process the probe/irrelevant stimuli. They simply press the single required probe/irrelevant button when something appears on the screen after the target/nontarget, without reading and processing that probe/irrelevant stimulus. Thus, for motivated subjects with life-changing consequences to the outcome, there is no processing of the information content of probes and irrelevants, there are no differences between probe and irrelevant ERPs, and accuracy rate is 0% for the four-stimulus complex trial protocol (Farwell et al., 2013). By contrast, the required button-press discrimination on every trial in the three-stimulus CIT ensures that the subjects read and process every stimulus, resulting in the predicted ERPs to targets, probes, and irrelevants and reliable results of the test. This behaviorally required button-press discrimination in response to every stimulus, including when a probe or irrelevant is presented, may not be necessary for tests with accommodating subjects and lacking any non-trivial consequences, as several studies (e.g., Rosenfeld et al., 2008; Dietrich et al., 2014; Meixner and Rosenfeld, 2014) have shown. It is, however, a necessary condition for reliable results in field use or any application when subjects are highly motivated not to reveal the concealed information, e.g., when they are facing major life-changing consequences to the outcome (Farwell et al., 2013).

#### Recent comparison-CIT publications provide additional evidence for necessary conditions

Several recent comparison-CIT studies have provided additional evidence for the necessary conditions for low error rates and high statistical confidences. Dietrich et al. (2014) conducted a four-stimulus comparison-CIT test and obtained results similar to those of other previous comparison-CIT studies, including ours. They varied the number of trials in the analysis, and concluded that “even procedures that utilize as few as 33 trials can reliably detect the presence of concealed information.” This is in accord with our finding that standard 13 is not a necessary condition. The statement that the four-stimulus complex trial protocol method of Dietrich et al. can “reliably detect…” depends on one's definition of “reliably.” Dietrich et al.'s results are indeed no less reliable than those of previous comparison-CIT studies. They are, however, much less reliable than the results obtained with the classification CIT in this research and all previous studies meeting the same standards. In separate experiments Dietrich et al. applied two different comparison CITs: the three-stimulus method and the four-stimulus method. Their subjects were accommodating college students who presumably read and processed the probe and irrelevant stimuli as instructed, despite the fact that the behavioral (button-press) demands of the task did not require them to do so in the four-stimulus method. Thus the probe and irrelevant waveforms were significantly different. They also found that subjects later recalled stimuli in both the three-stimulus and four-stimulus experiments. All of this is expected behavior and results for accommodating subjects when there are no non-trivial consequences to the outcome. Their study does not address the phenomenon described above that with the four-stimulus method, motivated subjects in situations with life-changing consequences to the outcome of the test do not read and process the probe and irrelevant stimuli because the button-press task does not require it in the four-stimulus method, and therefore the four-stimulus complex trial protocol has 100% error rate (0% accuracy) with highly motivated subjects and life-changing consequences to the outcome (Farwell et al., 2013).

Meixner and Rosenfeld (2014) conducted a comparison CIT. They published only bootstrap probabilities and failed to identify a specific criterion for distinguishing between information present (“knowledgeable”) and information absent (“nonknowledgeable”) subjects^4^. Applying the usual 90% criterion that was applied in all of their previous studies (and in virtually all others), Meixner and Rosenfeld's results are as follows. With one of their two analysis techniques, 25% of determinations for information-present subjects are false negatives, and only 33% of the information-absent determinations are valid, i.e., 67% are invalid, having a computed bootstrap probability of less than chance (50%) of being correct. Their other analysis technique correctly classifies only 33% of the information-present subjects, with 67% false negatives. (Any higher criterion would produce even more errors; any lower criterion would produce unacceptably low statistical confidences for both information-present and information-absent determinations). Their results contribute to establishing the necessary conditions for a viable ERP-based CIT. Their results are comparable to those of the other comparison-CIT studies published to date, including the research we report here. They provide additional data in support of the hypothesis that the application of the classification CIT, rather than the comparison CIT, is a necessary condition for obtaining low error rates, high statistical confidences, reliability, and validity.
